# Budget Impact Analysis of Treatment Flow Optimization in Epilepsy Patients: Estimating Potential Impacts with Increased Referral Rate to Specialized Care

**DOI:** 10.36469/jheor.2021.24061

**Published:** 2021-06-10

**Authors:** Masaki Iwasaki, Takashi Saito, Akiko Tsubota, Tatsunori Murata, Yuta Fukuoka, Kazutaka Jin

**Affiliations:** 1 Department of Neurosurgery, National Center Hospital, National Center of Neurology and Psychiatry; 2 Department of Child Neurology, National Center Hospital, National Center of Neurology and Psychiatry; 3 LivaNova PLC; 4 CRECON Medical Assessment. Inc; 5 CRECON Medical Assessment Inc.; 6 Department of Epileptology, Tohoku University Graduate School of Medicine

**Keywords:** referral rate, markov model, treatment flow optimization, epilepsy, budget impact

## Abstract

**Objectives:** We developed a Markov model to simulate a treatment flow of epilepsy patients who refer to specialized care from non-specialized care, and to surgery from specialized care for estimation of patient distributions and expenditures caused by increasing the referral rate for specialized care.

**Methods:** This budget impact analysis of treatment flow optimization in epilepsy patients was performed as a long-term simulation using the Markov model by comparing the current treatment flow and the optimized treatment flow. In the model, we simulated the prognosis of new onset 5-year-old epilepsy patients (assuming to represent epilepsy occurring between 0 and 10 years of age) treated over a lifetime period. Direct costs of pharmacotherapies, management fees and surgeries are included in the analysis to evaluate the annual budget impact in Japan.

**Results:** In the current treatment flow, the number of refractory patients treated with four drugs by non-specialized care were estimated as 8766 and yielded JPY5.8 billion annually. However, in the optimized treatment flow, the number of patients treated with four drugs by non-specialized care significantly decreased and who continued the monotherapy increased. The costs for the four-drug therapy by non-specialized care were eliminated. Hence cost-saving of JPY9.5 billion (-5% of the current treatment flow) in total national expenditures would be expected.

**Conclusion:** This study highlights that any policy decision-making for referral optimization to specialized care in appropriate epilepsy patients would be feasible with a cost-savings or very few budget impacts. However, important information in the decision-making such as transition probability to the next therapy or excuse for sensitive limitations is not available currently. Therefore, further research with reliable data such as big data analysis or a national survey with real-world treatment patterns is needed.

## INTRODUCTION

Although several new anti-seizure medications (ASMs) have been available for epilepsy treatment, 30% to 33% of patients remain resistant to medical treatment.^1–3^ Epilepsy surgery is the most important treatment option for drug-resistant epilepsy. Patients with drug-resistant epilepsy should be provided access to the appropriate specialty care leading to surgical treatment. However, the underutilization of epilepsy surgery has become an international issue since the number of epilepsy surgeries are much lower than expected and the situation has not changed even after the evidence on the efficacy of epilepsy surgery was established.^4,5^ The guidelines issued by the National Association of Epilepsy Centers pointed out that the appropriate referral process between non-specialist and specialist is the most difficult step in the journey of patients to surgical treatment.^6^ The community-based integrated care system for the drug-resistant epilepsy has not necessarily worked in each country due to individual situations.^7^

Recently, the World Health Organization recognized epilepsy as a major public health concern.^8^ The global campaign against epilepsy has been carried out by the World Health Organization, the International League Against Epilepsy, and the International Bureau for Epilepsy. The aim of the projects includes the reduction of the treatment gap in people with epilepsy and the development of models integrating epilepsy care into local health systems. The projects have shown that there are simple cost-effective ways to treat epilepsy in low-resource settings. One study demonstrated that epilepsy surgery could be performed within a resource-limited setting in low- to middle-income countries with the same quality as in high-income countries, so transfer of skills and expertise from high-income countries should be encouraged.^9^

Based on the National Database Open Data, the rate of epilepsy surgeries in patients with drug-resistant epilepsy in Japan is estimated at approximately half of that in the United States.^10^ The problem with Japan’s medical care system is that no medical department plays an initiative role in epilepsy care. Pediatricians, neurologists, neurosurgeons and psychiatrists have been independently involved in epilepsy care in Japan. This situation interferes with the acceleration of treatment cooperation. To solve the issues, the Ministry of Health, Labour and Welfare in Japan started in 2016 to build an epilepsy regional medical cooperation project. One of tactics is to assign one hospital as “the designated institution for epilepsy care” in each prefecture to improve patient flow to specialized care if the patient is considered drug-resistant.^11,12^

By the efforts of the parties, the designated institutions for epilepsy care were established in 21 prefectures as of December 2020. Additionally, the Japan Epilepsy Society initiated a comprehensive epilepsy center certification system in 2021. Efforts to improve regional medical cooperation into a national system are very unique in the world. Since the free-access system for primary care has been established in Japan, a truly optimal treatment flow can be established once access to specialized care increases. This structure defines an ideal model of medical cooperation in epilepsy care.

The aim of the study was to evaluate potential changes of nationwide patient distributions and budget impacts caused by increasing the epilepsy referral rate for specialized care in Japan. We expect that the information would contribute to recommendations for health-care policies in the future.

## METHODS

### Study Design

This budget impact analysis of treatment flow optimization in epilepsy patients was performed as a long-term simulation using the Markov model by comparing the current treatment flow and the optimized treatment flow. In the model, we simulated the prognosis of new onset 5-year-old epilepsy patients (assuming to represent epilepsy occurring between 0 and 10-years of age) treated over a lifetime period. Direct costs of pharmacotherapies, management fees and surgeries are included in the analysis to evaluate the annual budget impact in Japan.

**Figure 1.**
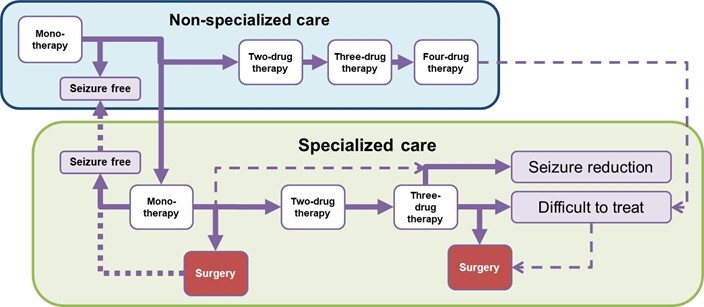
Simplified Model Structure

Patients were assumed to initiate treatment with monotherapy by non-specialized care and progress to combination therapy with second, third and fourth add-on drugs, with a certain probability when seizures were not controlled. Patients were assumed to transfer to specialized care at each treatment stage with a certain probability (see Figure 1 for the Simplified Model Structure and **Figure S1** in the supplementary appendix for the detailed model structure). From the Japanese public health-care payer’s perspective, only direct medical costs were included, and the discount rate was not applied to simply evaluate actual budget impact. The cycle length and time horizon of the analysis were adopted as one year and lifetime, respectively.

Table 1 shows the parameters in pharmacotherapy used in the model. The number of newly-diagnosed pediatric epilepsy patients were estimated based on the incidence of epilepsy for each age category and the Japanese population under 10 years of age.^13,14^ The incidence of epilepsy is not affected by lifestyle or race and no preventive measures are currently available for epilepsy. A budget impact analysis was also performed for the optimized treatment flow with increased referral rates to specialized care from the current condition.

**Table 1.** Clinical Parameters in Pharmacotherapy

	Current Treatment Flow	Optimized Treatment Flow	Source of Current Treatment Flow
Number of Newly-Diagnosed Pediatric Patients with Epilepsy Under 10 Years Old per Year	7117	7117	MHLW 2018, Aaberg 2017
Transition Probability to Next Therapy			
By Non-specialized Care			
From Monotherapy to Two-drug Therapy	38.6%	20.0%	Prescription database*
From Two-drug Therapy to Three-drug Therapy	27.1%	14.0%	
From Three-drug Therapy to Four-drug Therapy	23.5%	0.0%	
By Specialized Care			
From Monotherapy to Two-drug Therapy	27.0%	40.0%	Calibrated from 2019 data in Nagano prefecture
From Two-drug Therapy to Three-drug Therapy	57.0%	48.0%	
From Three-drug Therapy to Four-drug Therapy	77.0%	48.0%	
Transition Probability to Specialized Care from Non-specialized Care			
In Transition During Monotherapy	6.4%	20.0%	Calibrated from 2019 data in Nagano prefecture
In Transition During Two-drug Therapy	27.9%	56.0%	
In Transition During Three-drug Therapy	41.5%	90.0%	
Transition Probability to Surgical Treatment			
In Transition to Two-drug Therapy	3.0%	10.0%	Expert opinion
In Transition to Three-drug Therapy	3.0%	12.0%	
In Transition to Four-drug Therapy	3.0%	32.0%	
Annual Transition Probability in 40 Years after Transition to Four-drug Therapy/Difficult-to-treat			
To Specialized Care from Non-specialized Care	1.5%	-	Calibrated from 2019 data in Nagano prefecture
To Surgery from Specialized Care	1.5%	1.5%	
Annual Transition Probability in 5 Years from Specialized Care to Non-specialized Care (primary care)			
In Monotherapy	1.0%	80.0%	Calibrated from 2019 data in Nagano prefecture
In Two-drug Therapy	1.0%	50.0%	
In Three-drug Therapy	1.0%	20.0%	

*In house analysis from prescription data. Copyright © 2021 IQVIA. Calculated based on IQVIA Rx from January 2015 to June 2020. Reprinted with permission.

For the current treatment flow, the transition probabilities to the next therapy by non-specialized care were calculated by a prescription database provided by IQVIA Rx from January 2015 to June 2020 (The details regarding the real-world data analysis for the parameter estimation are described later). The transition probabilities to the next therapy by specialized care and the transition probability to specialized care from non-specialized care were calibrated to reproduce the annual number of surgeries from 2019 data of the Nagano prefecture for representing the current nationwide situation in Japan. The epilepsy center in the Nagano prefecture was only recently established and registered as the designated institution for epilepsy care in 2020. The transition probabilities to surgical treatment were assumed based on expert opinion. The annual probability of the transition from four-drug therapy in non-specialized care to difficult-to-treat state in specialized care, that of the transition from the difficult-to-treat state to surgery, and that of the transition from specialized care to non-specialized care (primary care) after achieving seizure freedom were also calibrated by 2019 data of the annual number of surgeries in the Nagano prefecture (Table 1). The former two transitions were considered over a period of 40 years after the onset of epilepsy, i.e., 5 years old, and the latter one was considered over a period of 5 years after achieving seizure freedom. Clinical parameters in surgery are shown in Table 2. We assumed that patients do not have a chance to improve their severity without the surgery, namely, to come back to previous health states in the model. All parameters for the optimized treatment flow were assumed based on expert opinion to reproduce the ideal situation for a true community-based integrated care system (see the **Supplementary File** for the detailed calculation and assumption for the estimated parameters). In the ideal scenario, the adherence to treatment guidelines was assumed to increase, resulting in the reduction of unnecessary combination therapy and the promotion of continued monotherapy. Transition probabilities to specialized care as well as to surgical treatment were also assumed to increase in order to provide early surgical treatment for patients with surgically remediable causes of drug-resistant epilepsy (**Supplementary File**).

**Table 2.** Clinical Parameters in Surgery

	Current Treatment Flow	Optimized Treatment Flow	Source of Current Treatment Flow
Probability of Transition to Surgical Treatment			
In Monotherapy			
Curative Surgery	49.0%	90.0%	National data base open data in Japan
Palliative Surgery	51.0%	10.0%	
In Two-drug Therapy			
Curative Surgery	49.0%	70.0%	National data base open data in Japan
Palliative Surgery	51.0%	30.0%	
In Three-drug Therapy			
Curative Surgery	49.0%	30.0%	National data base open data in Japan
Palliative Surgery	51.0%	70.0%	
Probability of Long-term Seizure Freedom after Surgery			
Curative Surgery	53.0%	53.0%	Expert opinion
Palliative Surgery	7.5%	7.5%	
Probability of Significant Seizure Reduction after Surgery			
Curative Surgery	73.0%	73.0%	Expert opinion
Palliative surgery	20.0%	20.0%	

Cost parameters for pharmacotherapy including management costs were calculated using the prescription database for non-specialized care and by real-world experience from the National Center Hospital, National Center of Neurology and Psychiatry, Tokyo, Japan for specialized care. The unit cost for each drug was based on the Japanese National Health Insurance list price and a dosage for the average Japanese weight by age category. Cost parameters for surgery were estimated by a standard of care for pharmacotherapies based on a patient’s weight by age category, and the Japanese medical fee list for surgeries (Table 3, Table 4).

**Table 3.** Cost Parameters for 3 Months Pharmacotherapy

	Pediatric (≤6 years of age)	Pediatric (>6 years of age)	Adult	Source
By Non-specialized Care				
Monotherapy	26 996	66 662	70 833	From Prescription data*
Two-drug Therapy	45 444	92 464	100 224	
Three﻿-﻿drug Therapy	68 014	121 571	131 913	
Four-drug Therapy	94 728	157 249	168 288	
By Specialized Care				
Monotherapy	35 623	79 938	88 609	National Center Hospital, National Center of Neurology and Psychiatry, Tokyo, Japan
Two-drug Therapy	53 635	103 440	116 485	
Three-drug Therapy	70 018	128 743	149 759	
In Seizure Reduction	85 151	140 941	150 628	
In Difficult-to-treat	85 151	140 941	150 628	

*In house analysis from prescription data. (Copyright © 2021 IQVIA. Calculated based on IQVIA Rx from October 2019 to June 2020.) Reprinted with permission.

**Table 4.** Cost Parameters for Surgery (JPY, estimated by standard of care)

	Value
≤9 Years of Age	2 023 280
10-14 Years of Age	2 001 008
15-19 Years of Age	2 079 145
Adult	2 102 324

The medical fee for surgery varies by patients’ age because the proportion of the types of surgeries performed depends on the patient’s age.

### Real-world Data Analysis for the Parameter Estimation

The transition probability to the next therapy and the cost parameters for pharmacotherapy by non-specialized care were estimated using the Japanese outpatient prescription database provided by IQVIA Rx from January 2015 to June 2020. ASM prescriptions were defined as the oral medications included in Anatomical Therapeutic Chemical Classification System code: N03A0, excluding analgesics and orphan drugs. The first date of ASM prescription was set as the index date if the patient had not been prescribed any ASMs for the previous six months, and the patients were followed over 36 months after the index date. The transition to the next therapy was defined as an addition of other ASMs in the same date prescription. The cost parameters for pharmacotherapy by non-specialized care were estimated by weighted average of real-world two-, three-, and four-drug combinations of the top eight frequently prescribed drugs in the database and three recently approved drugs. We excluded any combinations including other drugs from the calculation. Combinations of five or greater drugs were not considered.

The cost parameters for pharmacotherapy by specialized care were estimated using real-world data from the National Center Hospital, National Center of Neurology and Psychiatry, Tokyo, Japan. The combinations of ASMs considered were the same as above. The costs for seizure-free patients were calculated based on the prescribing patterns of patients who had been seizure-free for the previous year. The costs for seizure reduction and difficult-to-treat patients were based on the prescribing patterns of those with one or more seizures in the previous year.

### Sensitivity Analysis

To examine the robustness of the analysis result, one-way sensitivity analyses were performed. For the sensitivity analysis, +/- 20% of values for base-case analysis was employed for all parameters. We assumed that standard error of each parameter would be 0.1 of the point estimate and 95% confidence intervals were used for the ranges.

## RESULTS

The number of newly-diagnosed pediatric epilepsy patients under 10-years old in Japan was estimated to be approximately 7117/year of 0.57 million pooled population analyzed. The real-world treatment patterns in the prescription database and in the National Center Hospital, National Center of Neurology and Psychiatry were shown in **Table S1** and **Table S2**.

In the current treatment flow, the number of refractory patients treated with four drugs by non-specialized care were estimated as 8766 and yielded JPY5.8 billion annually. However, in the optimized treatment flow, the number of patients treated with four drugs by non-specialized care significantly decreased and who continued the monotherapy increased. Although the same trend was found, there were no big differences in the specialist care setting. The number of difficult-to-treat patients decreased and patients with seizure control substantially increased. The number of surgical treatments increased by 88%. Some 17.7% of surgeries (66 out of 372 surgeries) were performed in the early phase treatment, i.e. immediately after mono-, two-, or three-drug therapy, in the current treatment flow. The early phase surgeries increased to 81.7% (573 out of 701 surgeries) in the optimized treatment flow. The number of patients by non-specialized care setting decreased and that by specialized care setting increased, but the difference was not substantial. The costs for the four-drug therapy by non-specialized care were eliminated. Hence cost-saving of JPY9.5 billion (-5% of the current treatment flow) in total national expenditures would be expected (Table 5, Table 6).

**Table 5.** Results of Base-case Analysis for Patient Distribution

	Current Treatment Flow	Optimized Treatment Flow	Difference
Total	565 077	565 077	0
By Non-specialized Care	451 414	444 009	-7405
Monotherapy	321 701	388 862	67 161
Two-drug Therapy	99 713	51 008	-48 705
Three-drug Therapy	21 235	4139	-17 096
Four-drug Therapy	8766	0	-8766
By Specialized Care	113 291	120 366	7075
Monotherapy	23 966	25 449	1483
Two-drug Therapy	26 785	26 590	-195
Three-drug Therapy	12 537	11 013	-1524
Seizure Reduction	5717	22 411	16 694
Difficult-to-treat	44 285	34 902	-9383
Annual Number of Surgeries	372	701	329
In Monotherapy	14	142	128
In Two-drug Therapy	27	164	137
In Three-drug Therapy (immediately after transition)	25	267	242
In Successive Three-drug Therapy	305	127	-178

**Table 6.** Results of Base-case Analysis for Annual Cost (million JPY)

	Current Treatment Flow	Optimized Treatment Flow	Difference
**Total**	204 105	194 557	-9548
**By Non-specialized Care**	142 398	124 901	-17 497
Monotherapy	86 529	102 747	16 218
Two-drug Therapy	38 974	19 996	-18 978
Three-drug Therapy	11 050	2158	-8892
Four-drug Therapy	5846	0	-5846
**By Specialized Care**	48 892	41 144	-7748
Monotherapy	8339	8668	329
Two-drug Therapy	12 357	12 213	-144
Three-drug Therapy	7339	6425	-914
Seizure Reduction	0	5469	5469
Difficult-to-treat	20 857	8369	-12 488
**Surgery**	974	1733	759
In Monotherapy	32	329	297
In Two-drug Therapy	64	395	331
In Three-drug Therapy	879	1009	130
**After surgery**	11 450	24 868	13 418
Seizure Free	2314	5600	3286
Seizure Reduction	3284	6617	3333
Difficult-to-treat	5852	12 651	6799

The detailed results of the annual transition number of patients between each state in the simulation were shown in **Table S3**.

**Figure 2.**
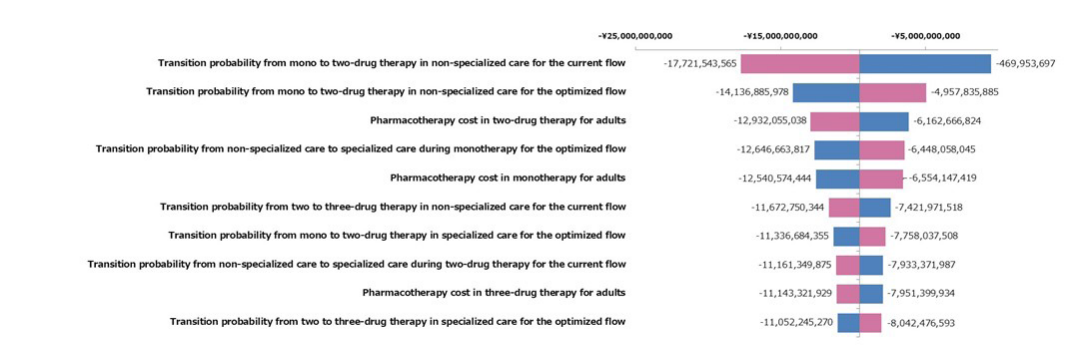
Tornado Diagram of One-way Sensitivity Analysis

One-way sensitivity analysis was performed with +/- 20% range of each parameter. The result of the one-way sensitivity analysis is presented as a tornado diagram in Figure 2. Although the point estimate of cost-saving in the base case analysis was JPY9.5 billion, there was no parameter to have a cost-increase potential in the worst range. If the most sensitive parameter for the incremental cost effectiveness ratio value, the transition probability from mono to two-drug therapy in non-specialized care for the current flow, was assumed to be the worst case, the budget impact calculation can be JPY-469 953 697.

## DISCUSSION

We developed a Markov model to simulate a flow of epilepsy patients who refer to specialized care from non-specialized care, and to surgery from specialized care for estimation of patient distributions and expenditures caused by increasing the referral rate for specialized care. Our model revealed that the number of patients in monotherapy and/or seizure free increased when the patient flow to specialized care was optimized. Decreased medical expenditures could be achieved by the optimization of patient flow.

The treatment flow optimization will increase the number of seizure-free patients with a cost-savings or a few negative budget impacts. The primary goal of epilepsy treatment is seizure control.^15^ It is important to achieve early seizure control with as few medications as possible and to consider early referral to specialty care when drug-resistance is suspected.^15^ A monotherapy is the most desirable and fundamental drug treatment for epilepsy.^16^ The role of specialized epilepsy care includes discrimination of “pseudo” drug-resistance, specialized drug adjustments such as the introduction of rational polypharmacy and the reduction of side effects, and surgical treatment.^15,17^ The number of patients with two- or three-drug therapy decreased and seizure free patients by surgery increased in the optimal flow. Difficult-to-treat patients can appropriately have access to specialized care and the timing of undergoing surgery is much faster than the current treatment flow—one of the primary reasons for the cost-savings from the treatment flow optimization. However, the results of the one-way sensitivity analysis suggested that the result would be significantly sensitive to the transition probability to the next therapy.

Surgical treatments should be provided for appropriate patients as early as possible. We could simulate that the treatment flow optimization would increase the number of surgical treatments and accelerate its early implementation. Drug-resistant epilepsy is a risk of premature mortality. The relative risk is five times that of the healthy population,^18^ but that is reduced 2.4 times when seizures are controlled by surgery.^19^ Surgical treatment reduces the number of patients with drug-resistant epilepsy. It has been reported that the shorter the disease duration, the better the postoperative seizure outcome by surgeries.^20^ Thus, it is important to provide surgical treatment at an appropriate time for patients with drug-resistant epilepsy.

The differences in treatment patterns between non-specialized care and specialized care were suggested by the analysis of the real-world treatment patterns.^21^ The prescription percentage of old-generation drugs was lower and that of new-generation drugs was higher in specialist care than in non-specialist care. Although there were many combination patterns for two- and three-drug therapy by non-specialized care, only a few optimized combinations were used by specialized care (**Table S1** and **S2**). Since the appropriate optimization of prescriptions is available by specialized care, we applied the increased proportion of patients who have seizure control with a small number of drugs by the acceleration of access to specialized care.

Specialized medical resources are limited, and efforts to return patients who do not need to continue specialized care to non-specialized care are important for effective use of the resources. As a result of this study, the number of patients pooled in specialized care has increased only slightly, and many patients whose seizures are controlled with a small number of drugs continue to be treated by non-specialists. There are only approximately 700 Board-certified epileptologists in Japan as of September 2020.^22^ Therefore, it is necessary to establish bidirectional cooperation that promotes not only access to specialized care but also counter-referrals to non-specialized care. Cost-savings of JPY9.5 billion in total national expenditures would be expected by treatment flow optimization. Although treatment costs of pharmacotherapy are slightly higher for specialized care, it was suggested that the total national expenditures could be reduced by providing appropriate treatment at the right time, rather than unnecessarily increasing specialists’ treatment.

This study implies that health-care policies in the early phase of treatment are critical for cost savings. Transition probabilities from mono- to two-drug therapy and from non-specialized to specialized care during monotherapy were sensitive to the result of budget impact (Figure 2). Several tactics should be considered to realize the optimized treatment flow. One is an educational act for non-specialists to increase adherence to treatment and referral guidelines. Another is providing reimbursement for the referral to specialized care. The latter is probably effective especially in countries with a universal health-care insurance system such as in Japan.

### Limitations

There are several limitations since the study is based on a model analysis. However, we believe that these do not affect the conclusion substantially. First, we did not include costs of adverse events by pharmacotherapy and by surgeries. The frequency and costs for adverse events are expected to decrease if specialized care is promoted. Second, pediatric self-limited epilepsy, which can spontaneously resolve by age, recurrent diseases, and re-operations were not considered in the analysis. However, these factors have the same influence on both the current and the optimal treatment flow, and, therefore, the impact on our conclusion would be limited. Third, the result was sensitive with several parameters such as transition probabilities to the next therapy based on the prescription database. The optimal setting was solely based on expert opinion. A wrong assumption in the sensitive parameters, such as the transition probability to specialized care during monotherapy (Figure 2), may significantly influence the results. Our conclusion would be uncertain if significant differences occur in multiple parameters between the current setting and the optimal setting. The estimated number of seizure-free patients in the pooled population was 65% and 72% in the current and the optimal settings, respectively (**Table S4**). These are close to the real-world picture reported in the previous study.^3,23^ Finally, our analysis only included direct medical cost as the outcomes. Seizure control by surgical treatment leads to improvement of a patient’s quality of life^24^ and social functions such as driving a car and employment. Therefore, the total societal benefit including not only direct medical costs but also indirect costs and patient’s well-being, etc., would be more considerable than our results.

## CONCLUSIONS

In conclusion, this study highlights that any policy decision-making for referral optimization to specialized care in appropriate epilepsy patients would be feasible with a cost-savings or very few budget impacts. However, important information in the decision-making process such as transition probability to the next therapy or excuse for sensitive limitations is not available currently. Therefore, further research with reliable data such as big data analysis or a national survey with real-world treatment patterns is needed.

### Disclosure

This study was sponsored by LivaNova PLC, Tokyo, Japan. Akiko Tsubota is an employee of LivaNova PLC. Tatsunori Murata is an employee of CRECON Medical Assessment Inc.

## Supplementary Material

Supplementary Materials
