# Supplementary material for: Budget Impact Analysis of Treatment Flow Optimization in Epilepsy Patients: Estimating Potential Impacts with Increased Referral Rate to Specialized Care
Source: J Health Econ Outcomes Res. 2021 Jun 10;8(1):80–7. doi: 10.36469/jheor.2021.24061 (PMC8192732; doi:10.36469/jheor.2021.24061)
Supplement: Supplementary Materials [file jheor_2021_8_1_24061_62942.pdf]

Journal of Health Economics and Outcomes Research

Supplementary Online Material

Iwasaki M, Saito T, Tsubota A, Murata T, Fukuoka Y, Jin K. Budget impact analysis of treatment flow optimization in epilepsy patients: estimating potential impacts with increased referral rate to specialized care. *JHEOR*. 2021;8(1):80-87. doi:10.36469/jheor.2021.24061

Supplemental Figure 1: Detailed Model Structure
Supplemental Table 1. Calculation from the Parameters Non-specialized Care
Supplemental Table 2. Calculation from the Parameters Specialized Care
Supplemental Table 3. Calculation from the Parameters Surgical Treatment

This supplementary material has been provided by the authors to give readers additional information about their work.

This is an open-access article distributed under the terms of the Creative Commons Attribution 4.0 International License (CCBY-4.0). View this license's legal deed at http://creativecommons.org/licenses/by/4.0 and legal code at http://creativecommons.org/licenses/by/4.0/legalcode for more information.

1. Probability of seizure freedom at each treatment phase by non-specialized and specialized care

Increased adherence to the guidelines will reduce unnecessary combination therapy and promote the continued monotherapy even by non-specialists.

-> The probability of seizure control will increase from 55% to 60% in monotherapy.

-> The probability of seizure control by the combination therapy decreases, because more patients will be managed by monotherapy and "truly difficult" patients will be subjected to the combination therapy. Similarly, the probability of seizure control in monotherapy at specialized care is reduced as "truly difficult" patients move to specialists at the early phase.

* With the optimized parameters we set (below), seizures are controlled with any medications in 80.2% of patients, with monotherapy in 70% of patients and with two-drug therapy in 9.2% of patients, respectively. Thus, 87.2% of seizure-free patients received monotherapy and 12.8% did the combination therapy.

* According to the literature by Stephen et al., 79.6% of seizure-free patients received monotherapy and 20.4% did the combination therapy[1]. In the long-term treatment, up to 30% of patients with newly-diagnosed epilepsy turn out to be drug-resistant.[2]

Supplemental Figure 1: Detailed Model Structure

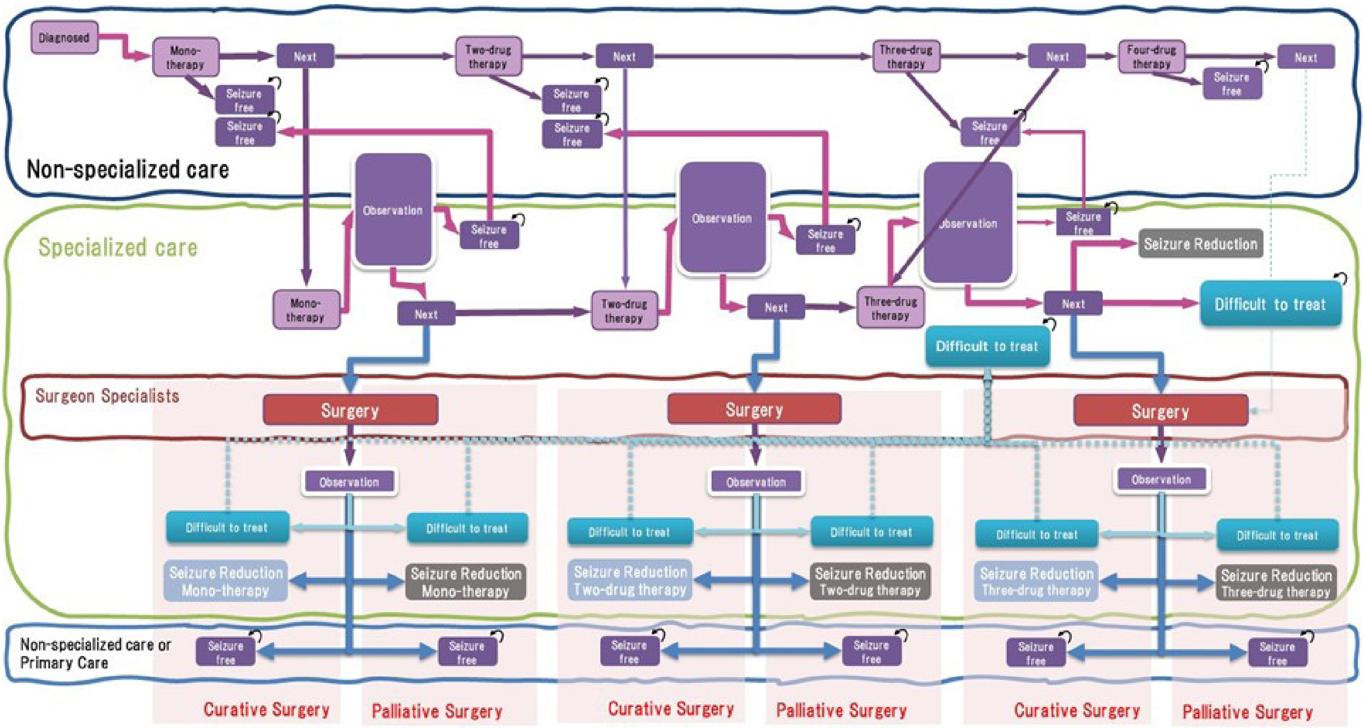

-> The above figure by the optimized treatment flow is better than, but within 10% difference compared to, the previous reports on the real-world data. We believe that the results are not too optimistic to realize.

Supplemental Table 1: Calculation from the Parameters Non-specialized Care

Seizure Control with Monotherapy	60%
Seizure Control with Two-drug Therapy	0.2 * 0.3 = 6%
Seizure Control with Three-drug Therapy	0.2 * 0.14 * 0.1 = 0.28%

Supplemental Table 2: Calculation from the Parameters Specialized Care	
Seizure Control with Monotherapy	0.2 * 0.5 = 10%
Seizure Control with Two-drug Therapy	0.2 * 0.4 * 0.4 = 3.2%
Seizure Control with Three-drug Therapy	0.2 * 0.4 * 0.48 * 0.2 = 0.768%

2. Transition probability to surgical treatments

The patients suitable for surgical treatment, i.e. those with surgically-remediable cause of epilepsy, undergo curative surgery at an early treatment phase. Palliative surgery remains as an important option for truly refractory patients, thus increasing indications at a relatively late treatment phase. Transition probabilities to specialized care as well as to surgical treatment are generally increased. Most patients should be referred to specialized care before the failure of three-drug therapy, giving a chance for surgical treatment.

-> The probability of curative surgery is high after mono- and two-drug therapy

-> The probability of palliative surgery is high after three-drug therapy

Hippocampal sclerosis, low-grade epilepsy associated brain tumors (LEAT), and vascular malformations are the three major causes of drug-resistant epilepsy, which are associated with high expectation of seizure freedom after surgical treatment. These account for 57.4% of all curative epilepsy surgeries.[3-4] Early surgical treatment is recommended for drug-resistant epilepsy with such surgically remediable etiologies.[5]

->These are considered as patients undergoing curative surgery during the therapy with mono- or two-drugs.

* With the optimized parameter sets below, 43.0% of surgery is provided after monotherapy and 16.0% of surgery is provided after two-drug therapy. Thus, 59% of epilepsy surgery will be performed before the failure of two-drug therapy.

-> This figure well demonstrates the ideal situation that patients with surgically remediable causes of drug-resistant epilepsy receive surgical treatment at early phase.

Supplemental Table 3: Calculation from the Parameters Surgical Treatment		
Curative Surgery after Monotherapy	0.2*0.1*0.9 = 1.8%	43.0%
Palliative Surgery after Monotherapy	0.2*0.1*0.1 = 0.2%	4.8%
Curative Surgery after Two-drugs Therapy	0.2*0.4*0.12*0.7 = 0.672%	16.0%
Palliative Surgery after Two-drugs Therapy	0.2*0.4*0.12*0.3 = 0.288%	6.9%
Curative Surgery after Three-drugs Therapy	0.2*0.4*0.48*0.32*0.3 = 0.369%	8.8%
Palliative Surgery after Three-drugs Therapy	0.2*0.4*0.48*0.32*0.7 = 0.860%	20.5%

Patients who subsequently undergo surgical treatment are not considered above.

REFERENCES

1. Stephen LJ, Forsyth M, Kelly K, Brodie MJet al. Antiepileptic drug combinations--have newer agents altered clinical outcomes? *Epilepsy Res*. 2012;98(2-3):194-198. doi:10.1016/j.eplepsyres.2011.09.008.
2. Chen Z, Brodie MJ, Liew D, Kwan P. Treatment outcomes in patients with newly diagnosed epilepsy treated with established and new antiepileptic drugs: a 30-year longitudinal cohort study. *JAMA Neurol*. 2018;75(3):279-286. doi:10.1001/jamaneurol.2017.3949.
3. Lamberink HJ, Otte WM, Blümcke I, et al. Seizure outcome and use of antiepileptic drugs after epilepsy surgery according to histopathological diagnosis: a retrospective multicentre cohort study. *Lancet Neurol*. 2020;19(9):748-757. doi:10.1016/S1474-4422(20)30220-9.
4. Blumcke I, Spreafico R, Haaker G, et al. Histopathological findings in brain tissue obtained during epilepsy surgery. *N Engl J Med*. 2017;377(17):1648-1656. doi:10.1056/NEJMoa1703784.
5. Engel J, Jr., McDermott MP, Wiebe S, et al. Early surgical therapy for drug-resistant temporal lobe epilepsy: a randomized trial. *JAMA*.012;307(9):922-930. doi:10.1001/jama.2012.220.
